# Higher mortality and impaired elimination of bacteria in aged mice after intracerebral infection with *E. coli* are associated with an age-related decline of microglia and macrophage functions

**DOI:** 10.18632/oncotarget.2709

**Published:** 2014-12-30

**Authors:** Sandra Schütze, Sandra Ribes, Annika Kaufmann, Anja Manig, Jörg Scheffel, Sandra Redlich, Stephanie Bunkowski, Uwe-Karsten Hanisch, Wolfgang Brück, Roland Nau

**Affiliations:** ^1^ Institute of Neuropathology, University Medical Center Göttingen, 37075 Göttingen, Germany; ^2^ Department of Geriatrics, Agaplesion Diakonissen Krankenhaus, 60322 Frankfurt am Main, Germany; ^3^ Department of Geriatrics, Evangelisches Krankenhaus Göttingen-Weende, 37075 Göttingen, Germany

**Keywords:** aging, bacterial CNS infection, phagocytosis, microglia, Toll-like receptor

## Abstract

Incidence and mortality of bacterial meningitis are strongly increased in aged compared to younger adults demanding new strategies to improve prevention and therapy of bacterial central nervous system (CNS) infections the elderly.

Here, we established a geriatric mouse model for an intracerebral *E. coli* infection which reflects the clinical situation in aged patients: After intracerebral challenge with *E. coli* K1, aged mice showed a higher mortality, a faster development of clinical symptoms, and a more pronounced weight loss. Elimination of bacteria and systemic inflammatory response were impaired in aged mice, however, the number of infiltrating leukocytes and microglial cells in the CNS of aged and young mice did not differ substantially.

*In vitro*, primary microglial cells and peritoneal macrophages from aged mice phagocytosed less *E. coli* and released less NO and cyto-/chemokines compared to cells from young mice both without activation and after stimulation by agonists of TLR 2, 4, and 9.

Our results suggest that the age-related decline of microglia and macrophage functions plays an essential role for the higher susceptibility of aged mice to intracerebral infections. Strategies to improve the phagocytic potential of aged microglial cells and macrophages appear promising for prevention and treatment of CNS infections in elderly patients.

## INTRODUCTION

One major health issue arising with age is the increasing prevalence and severity of infectious diseases [1, 2]. Young adults have a low risk of acquiring and an even lower risk of dying from bacterial infections. In later adulthood, the relative frequency of many bacterial infections, including pneumonia, urinary tract infections, sepsis and meningoencephalitis increases with age reflecting a weakening of the immune system in old individuals [e.g., 3, 4].

In persons ≥ 60 years, the incidence of *Streptococcus pneumoniae* meningitis is approximately 4 times higher and the relative frequency of *Listeria monocytogenes* meningitis is even 15 times higher compared to persons from 2–29 years of age [5]. Moreover, the outcome after bacterial CNS infections in old individuals is much worse than in young persons, and death in the acute phase of the disease and neurological or neuropsychological sequelae are frequent complications [6]. Therefore, there is an increasing need to identifiy strategies that can protect the elderly against bacterial CNS infections. To develop preventive or therapeutic strategies, the underlying mechanisms leading to the increased susceptibility of the elderly to bacterial CNS infections have to be identified.

The changes occurring in the immune system of the aging individual are extensive and affect both the adaptive and innate immune response. Old individuals produce less antibodies against epitopes of pathogens, and the specificity of antibodies declines, whereas the production of autoantibodies increases with age [7]. The density and proliferation of naïve B- and T-lymphocytes is reduced. The phagocytic capacity, release of cyto-/chemokines and reactive oxygen species and the intracellular killing of pathogens by granulocytes and macrophages are impaired in old individuals [8, 9, 10].

In CNS, meningeal and perivascular macrophages and microglia represent the first line of defense against bacteria. Microglial cells are the major constituents of the innate immunity in the CNS parenchyma [11]. Parenchymal microglia as well as meningeal and perivascular macrophages which become activated by bacterial products are critically involved in protecting the brain from infection [12]. *In vitro*, after exposure to bacteria or bacterial products, microglial cells release TNFα, MIP-2, IL-10, and nitric oxide, and exhibit antibacterial activity against *Streptococcus pneumoniae* and *Escherichia coli* [13, 14, 15, 16, 17]. Whereas microglial cells exert protective effects by phagocytosis of pathogens and cell debris and mediate repair mechanisms, their overstimulation can lead to a destruction of neuronal axons and somata [18, 19, 20].

Macrophages and microglial cells express Toll-like receptors (TLR) which are part of the innate immune system and recognize a variety of pathogens and pathogen-products [21, 22]. TLR on microglia are stimulated during the early phase of CNS infections and systemic infections [23, 24, 13, 25]. Especially important for bacterial infections are TLR2, TLR4 and TLR9: TLR2 is activated by bacterial lipopeptides, TLR4 recognizes endotoxin (LPS) and pneumolysin (an important pathogenic factor of *S. pneumoniae)*, and TLR9 is activated by bacterial DNA [21, 26, 13]. After activation by a ligand, TLR signaling leads to the production of inflammatory cytokines via MyD88-dependent and MyD88–independent pathways. Unlike in frailty, in healthy aging the expression of TLR on the surface of phagocytes appears not to decrease [27, 28, 29, 30]. At present, however, it is unknown, whether microglia in old age behave in the same way as macrophages and lose their ability to phagocytose and kill pathogens or whether their function is unaffected by age. For these reasons, in the present study, we compared the ability of young and aged microglial cells to phagocytose and kill bacteria as well as the course of *E. coli* meningitis induced by inoculation of bacteria into the CNS in young and healthy aged mice.

## RESULTS

### Higher mortality, faster development of clinical symptoms, and more pronounced weight loss in aged mice after intracerebral infection with *E. coli* K1

22 aged mice (26.2 ± 2.3 months) and 26 young mice (2.2 ± 0.3 months) received an intracerebral injection containing 1 × 10^5^ CFU *E. coli* K1. During the acute phase of the infection [up to 96 hours post infection (p.i.)] mice were monitored every 12 hours, and then on day 7, 10, 12, and 15 p.i. Monitoring of the mice included weighing and assignment of a clinical score.

**Figure 1:**
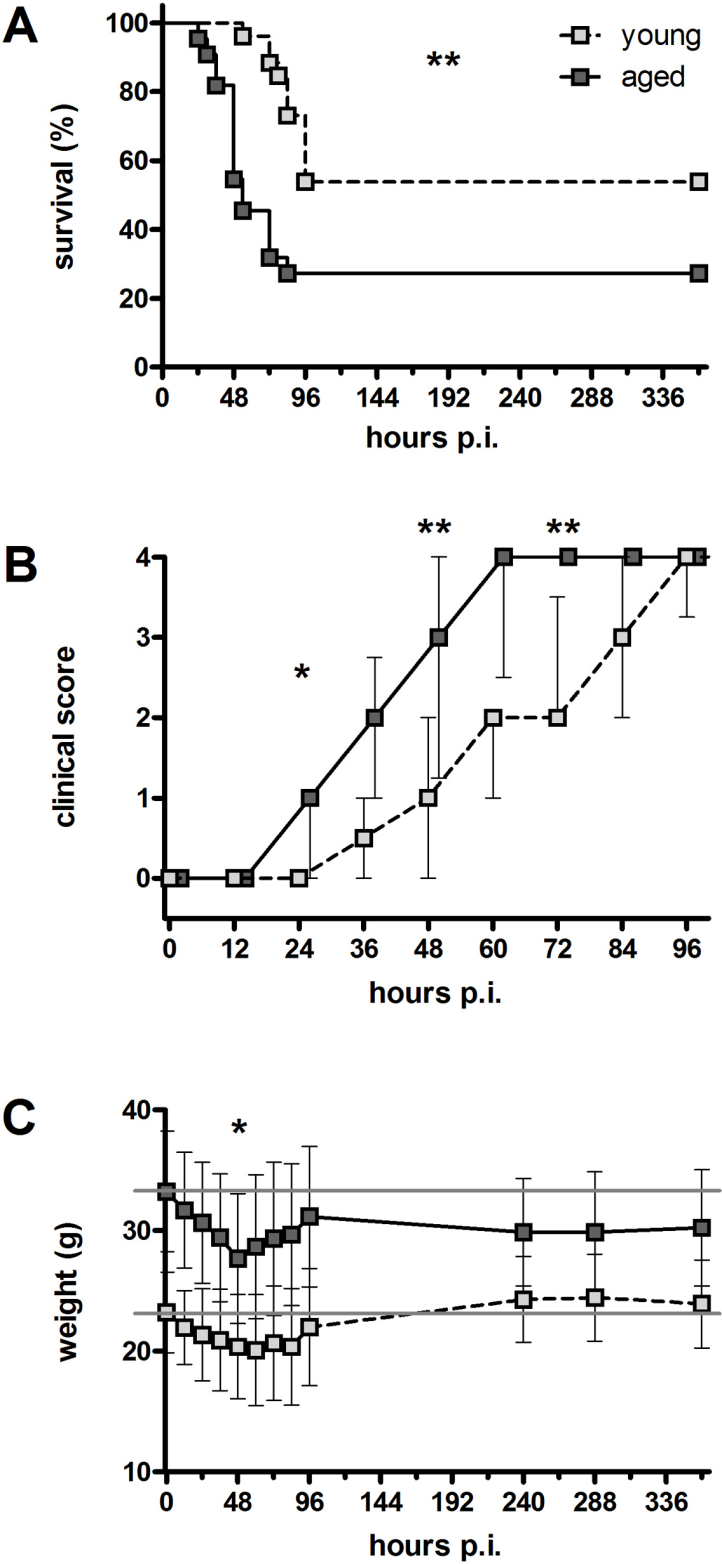
Clinical disease course after an intracerebral infection with *E. coli* K1 (10^5^ CFU/ml) in young and aged mice **(A)** Mortality following intracerebral *E. coli* K1 infection was significantly higher in aged mice (73%) than in young mice (47%; **p = 0.0025, log-rank test). **(B)** Deceased aged mice showed a significantly higher clinical score than deceased young mice 24 hours p.i. (*p = 0.02), 48 hours p.i. (**p = 0.008), and 72 hours p.i. (**p = 0.002; Mann-Whitney *U*-test followed by Bonferroni correction) indicating a faster development of clinical symptoms. Data are shown as medians (25./75. percentile). **(C)** Weight loss 48 hours p.i. was more pronounced in aged mice than in young mice (4.7 ± 2.5 g versus 2.8 ± 2.2 g, *p = 0.02; Student's *t*-test). After the acute phase of the infection, young mice rapidly gained weight again, whereas aged mice did not recover weight. Data are shown as means ± SD.

Mortality of aged mice was significantly increased compared to young mice: 16 of 22 aged mice (73%) died within 24–84 hours p.i (median = 48 hours), but only 12 of 26 young mice (47%) died within 48–96 hours p.i. (median = 84 hours) (log-rank test: *p* = 0.0025; Figure 1A). Surviving mice were sacrificed 15 days p.i..

Aged mice showed a faster development of clinical symptoms compared to young mice: the clinical score of deceased aged mice was significantly higher than the clinical score of deceased young mice 24 hours p.i. (*p* = 0.02), 48 hours p.i. (*p* = 0.008), and 72 hours p.i. (*p* = 0.002; Figure 1B).

Before infection, the weight of aged mice (33.3 ± 5.0 g; *n* = 22) was significantly higher than the weight of young mice (23.2 ± 3.4 g; *n* = 26; *p* < 0.0001). Both aged and young mice significantly lost weight during the acute phase of the infection: At 48 hours p.i., the weight of aged mice was 27.7 ± 5.4 g (*n* = 17; *p* = 0.002 compared to their weight before infection), and the weight of young mice was 20.4 ± 4.4 g (*n* = 26; *p* = 0.011 compared to their weight before infection). In aged mice, this weight loss was more pronounced than in young mice (4.7 ± 2.5 g versus 2.8 ± 2.2 g, *p* = 0.02). After the acute phase of the infection, young mice rapidly gained weight again: At 10 days p.i. weight of young mice was 24.3 ± 3.6 g (*n* = 14; *p* = 0.007 compared to their weight at 48 hours p.i.), even slightly higher than the pre-infection values. However, aged mice did not gain weight again after the acute phase of the infection: At 10 days p.i. weight of aged mice was 29.9 ± 4.4 g (*n* = 6; *p* = 0.38 compared to their weight at 48 hours p.i.; Figure 1C).

### Faster systemic spread of infection and decreased elimination of *E. coli* in aged mice

**Figure 2:**
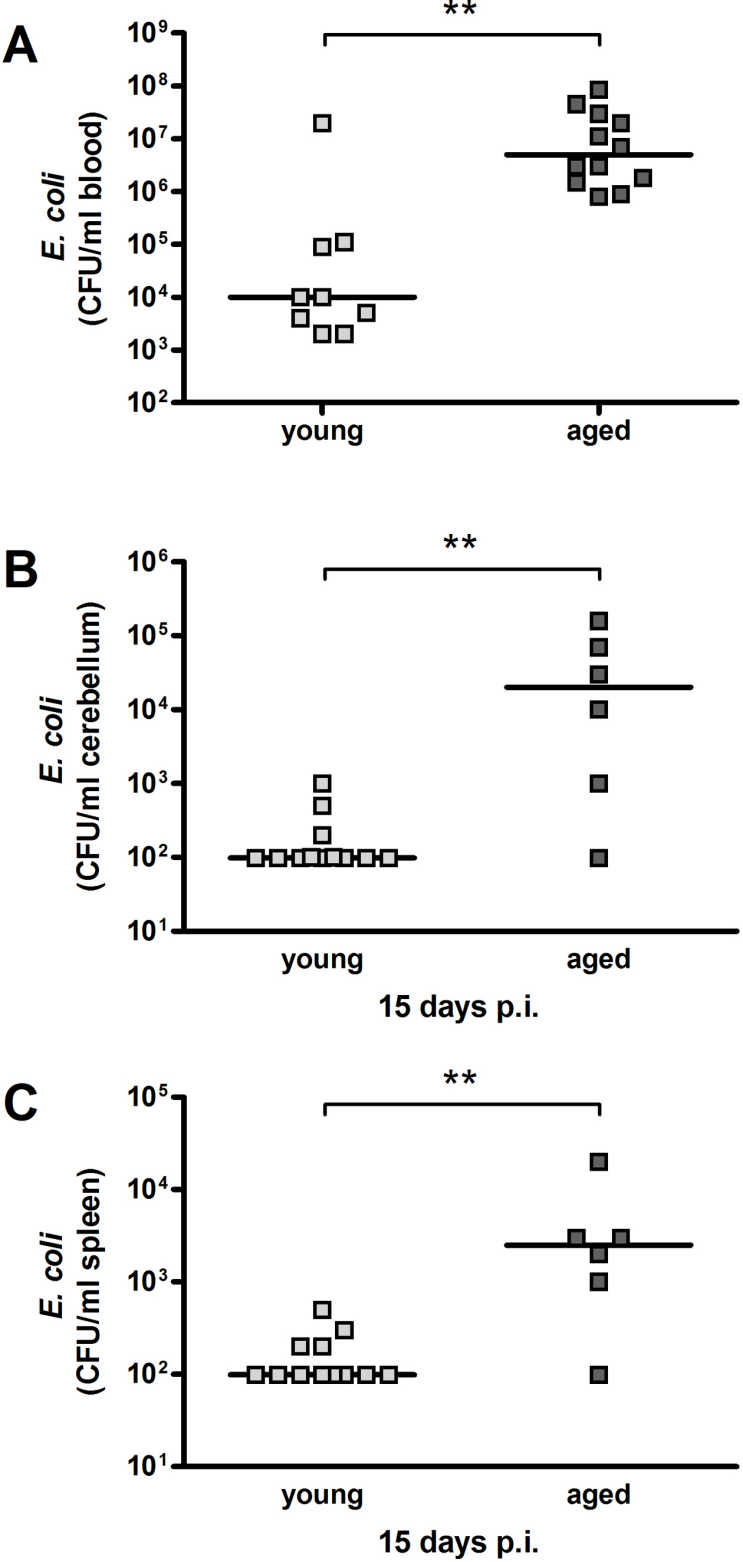
Bacterial concentrations in blood, cerebellum and spleen after intracerebral infection with *E. coli* K1 (10^5^ CFU/ml) in young and aged mice *E. coli* concentrations (CFU/ml) were significantly higher in aged compared to young mice in blood at the time of death due to infection (**p = 0.0014) **(A)** as well as in cerebellum (**p = 0.009) **(B)** and spleen (**p = 0.006) **(C)** 15 days p.i. (Mann-Whitney *U*-test). Symbols represent individual mice and bars indicate median values.

At 24 hours p.i., 62% of aged mice (8 of 13) but only in 36% of young mice (5 of 14) had positive blood cultures (detection limit 1000 CFU/ml), however, the difference between the concentrations of *E. coli* in blood of aged and young mice was not statistically significant [3000 (<1000/9500) versus <1000 (<1000/4000) CFU/ml; *p* = 0.12]. At the time of death as a consequence of infection, aged mice (*n* = 12) had significantly higher bacterial loads in blood than young mice (*n* = 9) [5 × 10^6^ (1.6 × 10^6^/1.8 × 10^7^) versus 1 × 10^4^ (3 × 10^3^ /1 × 10^5^) CFU/ml; *p* = 0.0014; Figure 2A]. Bacterial concentrations in cerebellum did not differ between old (*n* = 16) and young (*n* = 12) mice at the time of death due to infection [2.8 × 10^7^ (6 × 10^6^/1.2 × 10^8^) versus 5 × 10^7^ (2.4 × 10^7^/8.9 × 10^7^) CFU/ml; *p* = 0.46]. In spleen, bacterial concentrations at the time of death due to infection were slightly higher in aged mice [1 × 10^7^ (1.0 × 10^6^/8.5 × 10^7^), *n* = 16] than in young mice [1.9 × 10^6^ (1.3 × 10^5^ /9.3 × 10^6^) CFU/ml; *n* = 12], however, the difference was not statistically significant (*p* = 0.095).

At 15 days p.i., bacterial concentrations in cerebellum and spleen were significantly higher in surviving old mice (*n* = 6) than in surviving young mice (*n* = 14) [cerebellum: 20000 (775/92500) versus <100 (<100/175) CFU/ml; *p* = 0.009; Figure 2B / spleen: 2500 (775/7250) versus <100 (<100/200) CFU/ml; *p* = 0.006; Figure 2C]. In 7 of 14 young mice (50%) but only in 1 of 6 aged mice (17%), no bacteria were detected in cerebellum and spleen (detection limit 100 CFU/ml).

### Reduced serum levels of IL-6 and KC in aged mice in the acute phase of *E. coli* meningitis

In order to compare the concentrations of the pro-inflammatory cytokines TNF-α, IL-6, and the chemokine KC in cerebellum homogenates and serum of aged and young mice in the acute phase of infection, 36 aged (18.8 ± 0.9 months) and 37 young mice (2.8 ± 0.3 months) were intracerebrally infected with a higher dose of *E. coli* K1 (7.5 × 10^5^ CFU/mouse) and sacrificed 24 hours later. At this time, the clinical score of aged mice was significantly higher than that of young mice [1 (0/2) versus 0 (0/1); *p* = 0.0001].

Cerebellum concentrations of TNF-α, IL-6, and KC did not differ between aged and young mice 24 hours p.i. [TNF-α: 27 (18/36) pg/ml versus 30 (18/53) pg/ml, *p* = 0.46; IL-6: 433 (231/582) pg/ml versus 386 (226/618) pg/ml, *p* = 0.67; KC: 891 (574/1726) pg/ml versus 988 (620/1399) pg/ml, *p* = 0.86].

**Figure 3:**
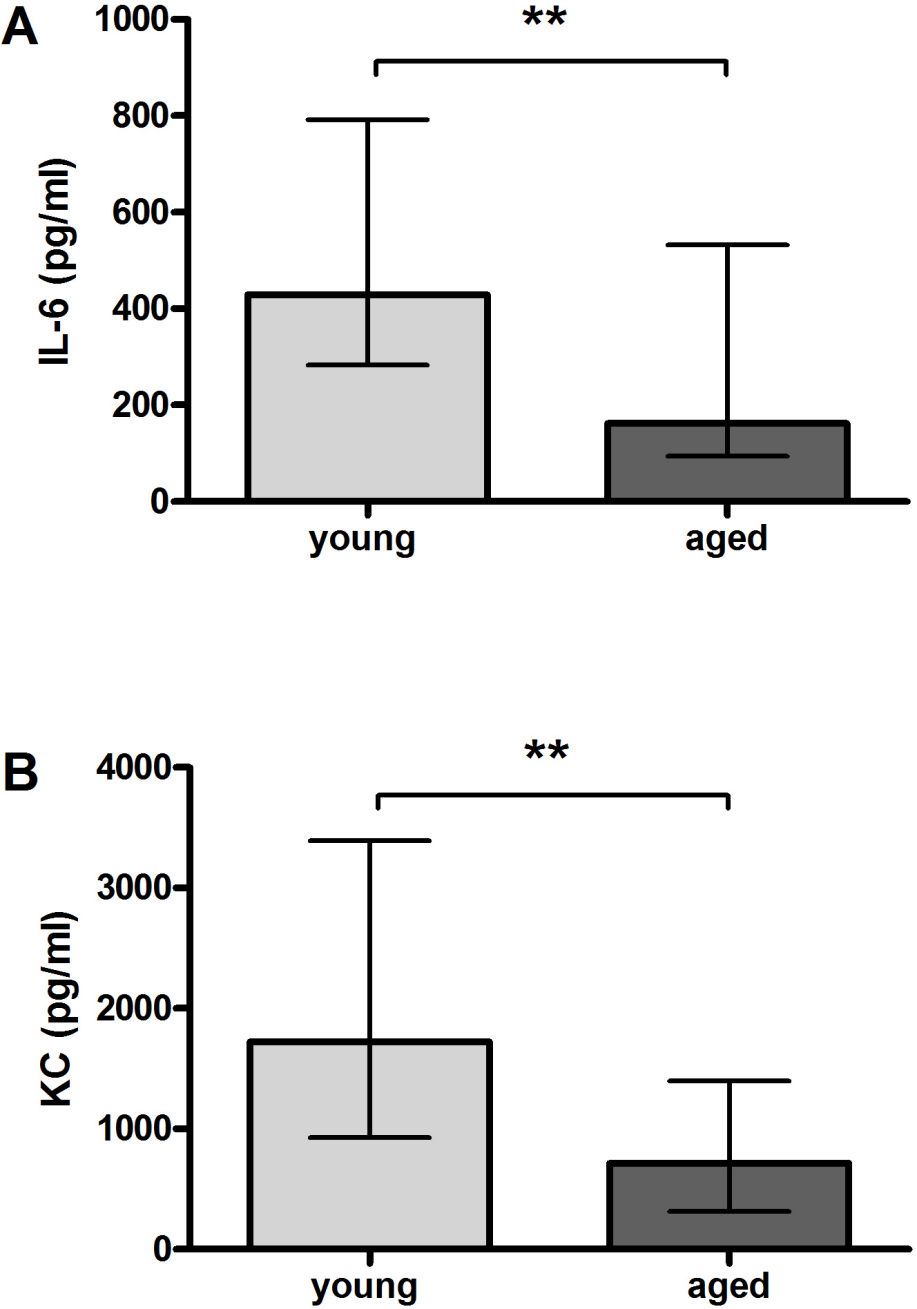
Serum concentrations of pro-inflammatory cyto-/chemokines after intracerebral infection with *E. coli* K1 (7.5 × 10^5^ CFU) in young and aged mice Serum levels of IL-6 (**p = 0.007) **(A)** and KC (**p = 0.002) **(B)** were significantly lower in aged than in young mice 24 hours p.i. (Mann-Whitney *U*-test). Data are shown as medians (25./75. percentiles).

Serum concentrations of IL-6 and KC 24 hours p.i. were significantly lower in aged mice compared to young mice reflecting a reduced systemic inflammatory response in aged mice during the acute phase of infection [IL-6: 162 (94/532) pg/ml versus 428 (283/792) pg/ml, *p* = 0.007; Figure 3A; KC: 714 (315/1399) pg/ml versus 1726 (929/3390) pg/ml, *p* = 0.002; Figure 3B]. Serum concentrations of TNF-α were below the limit of detection in both groups.

### Strong correlation of leukocyte invasion into the CNS and bacterial concentrations in cerebellum in both aged and young mice

**Figure 4:**
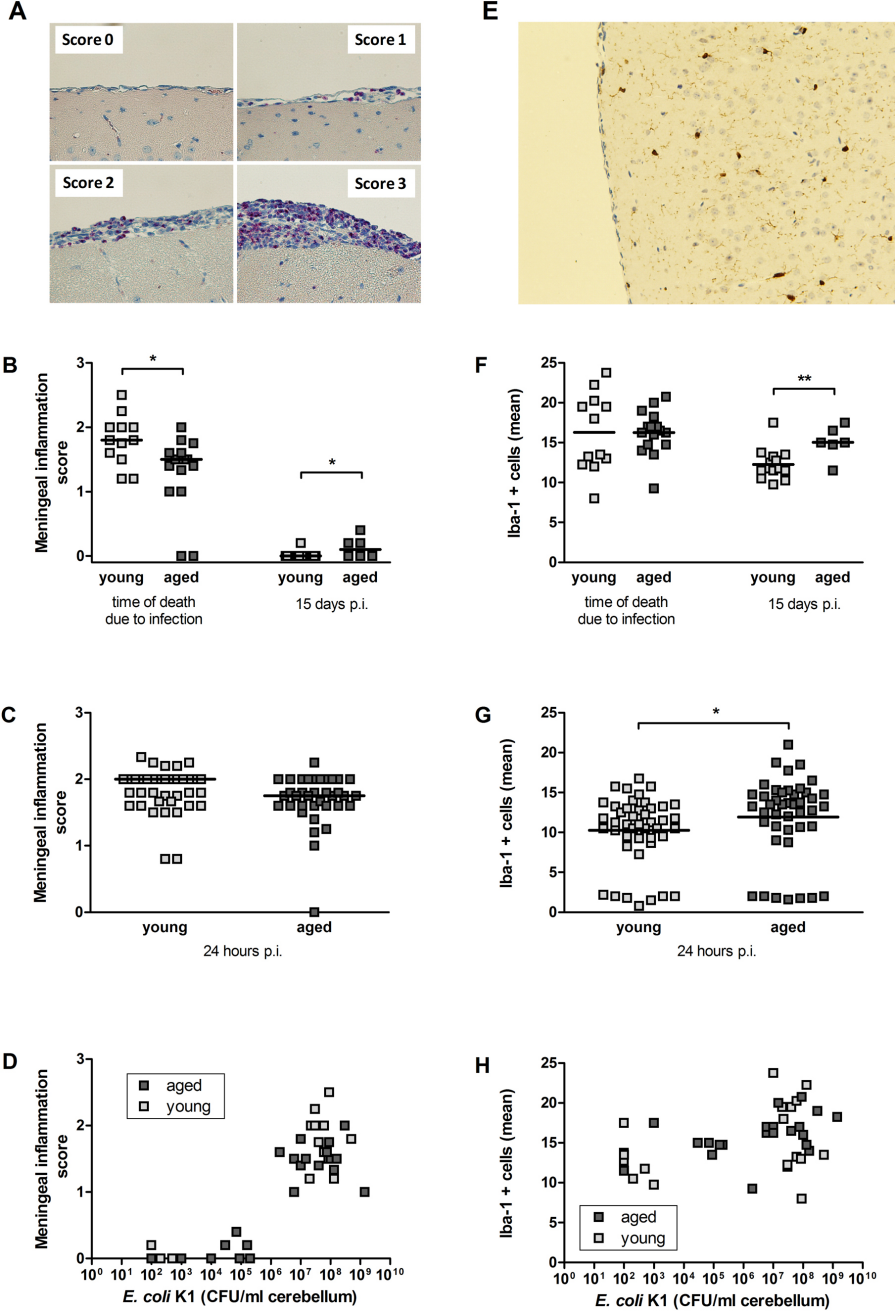
Quantification of infiltrating leukocytes (A–D) and microglial cells (E–H) in the CNS after intracerebral infection with *E. coli* K1 in young and aged mice **(A)** Illustrative examples of meningeal inflammation scores in chloroacetate esterase (CAE) stainings of brain sections after intracerebral challenge with *E. coli* K1. CAE-positive cells in pink represent infiltrating leukocytes, magnification × 40. **(B)** Meningeal inflammation scores after intracerebral challenge with 1 × 10^5^ CFU/ml were significantly lower in aged compared to young deceased mice at the time of death as a consequence of infection (*p = 0.01) but significantly higher in aged compared to young surviving mice 15 days p.i. (*p = 0.03, Mann-Whitney *U*-test). Symbols represent individual mice and bars indicate median values. **(C)** Meningeal inflammation scores of young and aged mice 24 hours after intracerebral challenge with 7.5 × 10^5^ CFU/ml did not differ. Symbols represent individual mice and bars indicate median values. **(D)** The meningeal inflammation score strongly correlated with the amount of bacteria in the CNS of both aged (r_s_ = 0.71; *p* = 0.0002) and young mice (r_s_ = 0.78; *p* < 0.0001). **(E)** Illustrative example of an Iba-1-stained brain section after intracerebral challenge with *E. coli* K1. Iba-1^+^ cells in brown represent microglial cells, magnification × 20. **(F)** Numbers of Iba-1^+^ cells after intracerebral challenge with 1 × 10^5^ CFU/ml did not differ between aged and young deceased mice at the time of death due to infection but were significantly higher in aged compared to young surviving mice 15 days p.i. (**p = 0.0095, Mann-Whitney *U*-test). Symbols represent individual mice and bars indicate median values. **(G)** Numbers of Iba-1+ cells in young and aged mice 24 hours after intracerebral challenge with 7.5 × 10^5^ CFU/ml were significantly higher in aged compared to young mice (*p = 0.015, Mann-Whitney *U*-test). Symbols represent individual mice and bars indicate median values. **(H)** Numbers of Iba-1^+^ cells did not correlate with bacterial concentrations in the CNS of both aged and young mice.

During bacterial meningitis, circulating leukocytes, predominantly neutrophilic granulocytes and monocytes, quickly enter the subarachnoid space as key components of the innate immune response. Chloroacetate esterase (CAE) staining was performed on brain sections of all mice in order to identify infiltrating neutrophilic granulocytes and to assess meningeal inflammation. Representative examples of the degrees of meningeal inflammation (scores 0–3) in CAE-stained brain sections are shown in Figure 4A.

Aged mice that died during the acute phase of the infection (24–78 hours p.i.) showed less infiltrating leukocytes than young mice that died during the acute phase of the infection (48–96 hours p.i.) reflected by a lower meningeal inflammation score [1.5 (1.1/1.6) versus 1.8 (1.5/2), *p* = 0.01; Figure 4B]. Aged mice that survived the infection and were sacrificed 15 days p.i. had a higher meningeal inflammation score than young mice that were sacrificed 15 days p.i. [0.1 (0/0.3) versus 0 (0/0), *p* = 0.03; Figure 4B], demonstrating a weak but ongoing meningeal inflammation in aged mice that was absent in young mice which were able to clear the infection. Assessment of meningeal inflammation in aged and young mice that were sacrificed 24 hours after intracerebral infection with a higher dose of *E. coli* K1 revealed that the number of infiltrating leukocytes at this defined point of time during the acute phase of the infection did not differ significantly between aged and young mice [meningeal inflammation score 1.75 (1.60/2) versus 2 (1.63/2), *p* = 0.07; Figure 4C]. The meningeal inflammation score strongly correlated with the bacterial concentrations in the CNS in both aged mice (Spearman's rank correlation coefficient r_S_ = 0.71, *p* = 0.0002) and young mice (r_S_ = 0.78, *p* < 0.0001; Figure 4D).

### No reduction of microglia numbers in aged mice *in vivo*

We evaluated cortical regions of Iba-1-stained brain sections to quantify microglial cells of young and aged mice after intracerebral infection with *E. coli* K1. Representative examples of Iba-1^+^ cells are shown in Figure 4E. The number of Iba-1^+^ cells did not differ between young and aged mice that died during the acute phase of the infection (*p* = 0.99; Figure 4F). The number of Iba-1^+^ cells in brains of aged mice that survived the infection and were sacrificed 15 days p.i. was higher than in young mice that were sacrificed 15 days p.i. (*p* = 0.0095; Figure 4F). 24 hours after intracerebral infection with a higher dose of *E. coli* K1, the number of Iba-1^+^ cells was slightly but significantly higher in aged mice than in young mice (*p* = 0.015; Figure 4G). There was no correlation between the numbers of Iba-1^+^ cells and the bacterial concentrations in the CNS in both aged and young mice (Figure 4H).

### No morphological differences between peritoneal macrophages and microglial cells from aged and young mice *in vitro*

Peritoneal macrophages and primary microglial cells from aged (18 months) and young mice (2 months) were used to analyze age-associated changes of macrophage and microglia functions *in vitro*.

The number of macrophages that could be obtained by lavage from the peritoneal cavity was considerably higher in aged than in young mice [159 ± 105 × 10^5^ (*n* = 9) versus 33 ± 18 × 10^5^ (*n* = 9); *p* = 0.003].

Macrophages and microglia were characterized and compared regarding their morphology, their ability to phagocytose *E. coli* K1, as well as their release of NO and different cyto-/chemokines both without activation and after stimulation by different TLR agonists. Due to the limited amount of microglial cells that could be prepared from the adult brains, only selected concentrations of TLR agonists were used for stimulation of microglial cells.

**Figure 5:**
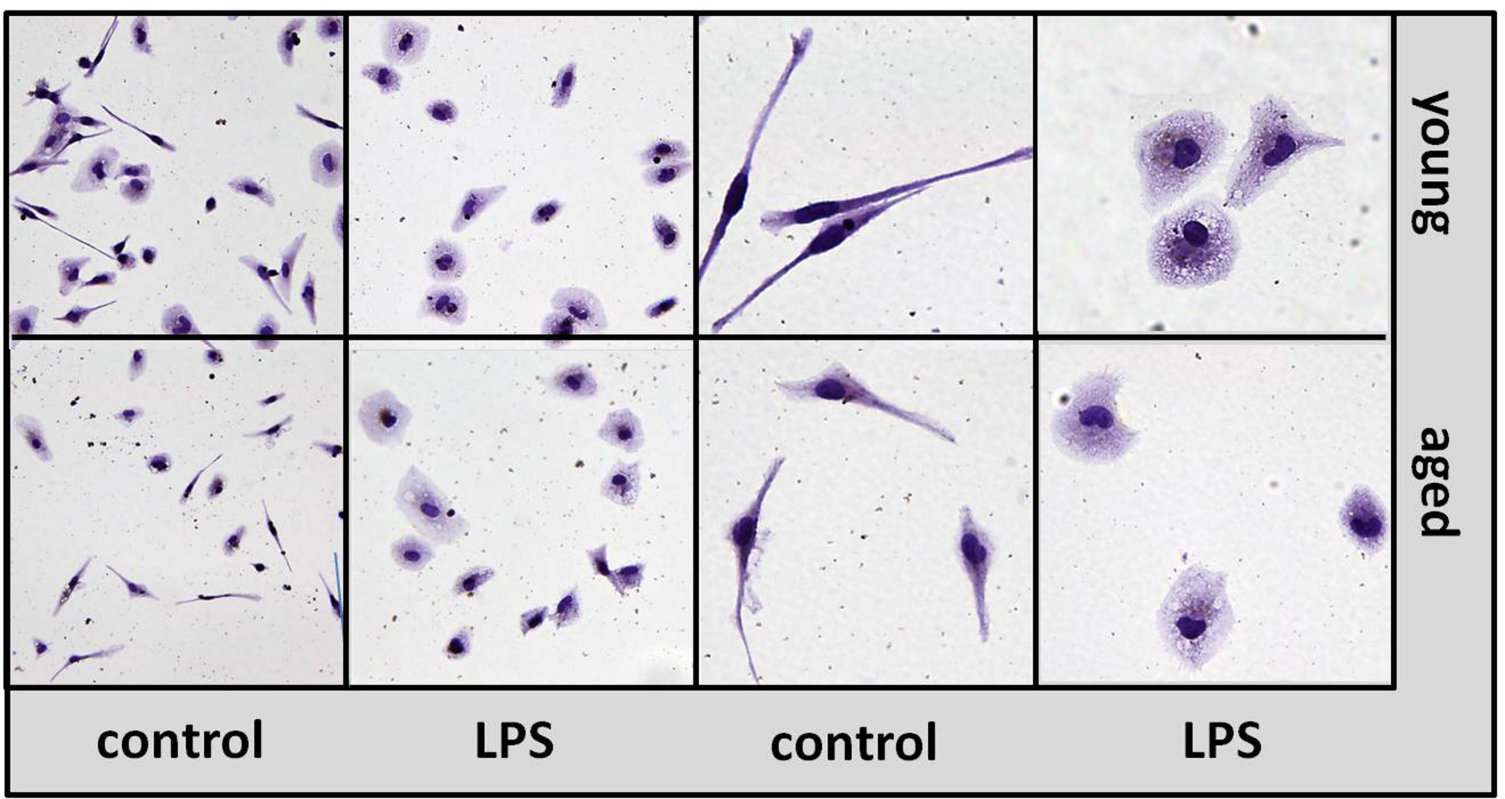
Morphology of macrophages from aged and young mice *in vitro* Isolectin B4 staining of macrophages prepared from young (upper panel) and aged (lower panel) mice in non-activated state (control) and after treatment with LPS 1 μg/ml for 24 hours (LPS). Left panels: magnification x 20, right panels: magnification x 40.

Morphologically, no differences were detected between macrophages from young and aged mice (Figure 5) and between microglial cells from young and aged mice (not shown), neither in the non-activated nor in the activated state. Vitality of the cells was additionally checked using the WST-1 assay (data not shown). Treatment with the different TLR agonists at the highest concentration of each TLR agonist that was used in the stimulation experiments did not influence the vitality of young or aged macrophages and microglial cells.

### Impaired phagocytosis of *E. coli* K1 by aged macrophages and microglial cells *in vitro*

**Figure 6:**
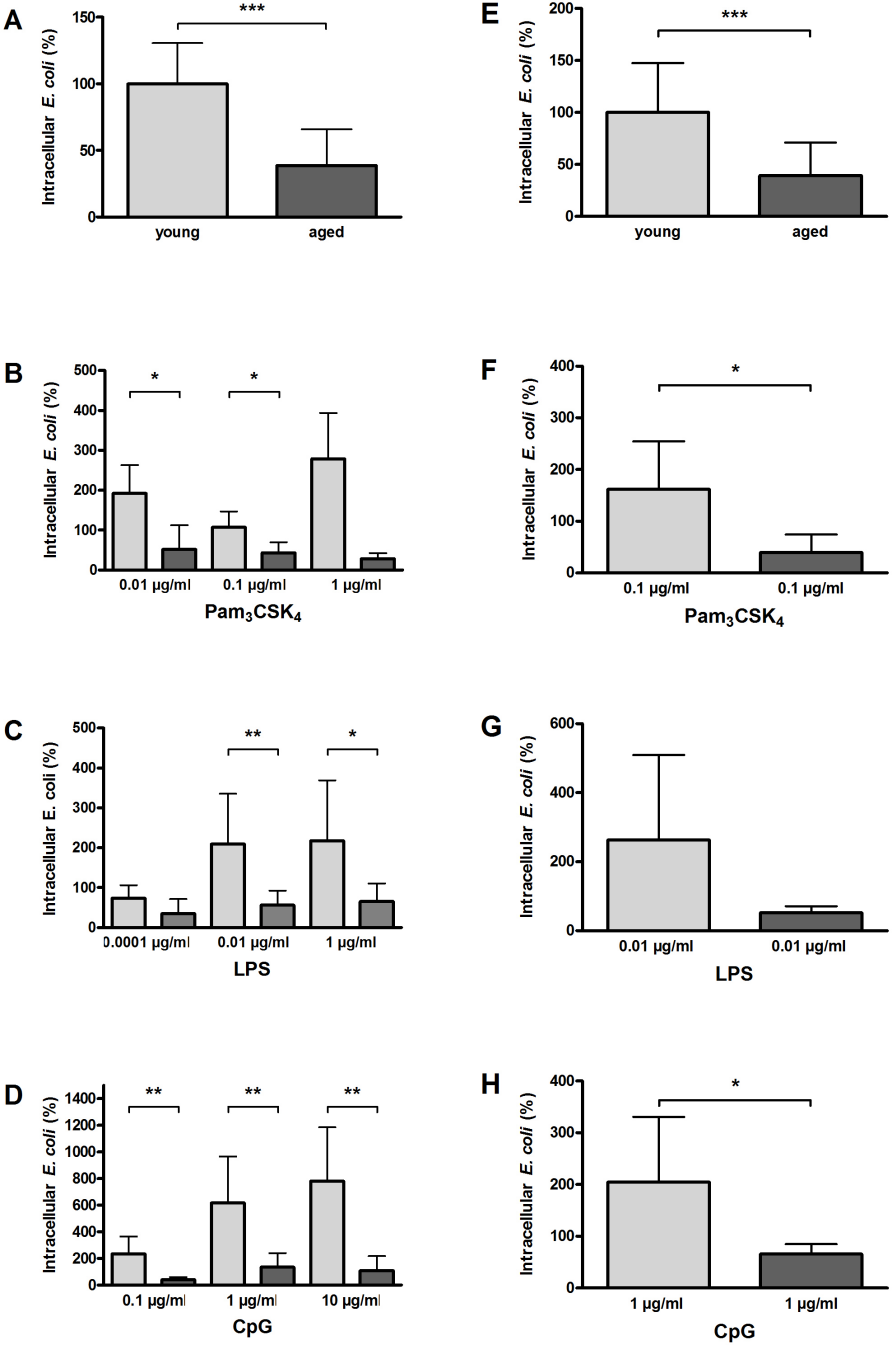
Phagocytosis of *E. coli* K1 by macrophages (A–D) and microglial cells (E–H) from young and aged mice *in vitro* In non-activated state, phagocytosis of *E. coli* was significantly lower in aged compared to young macrophages (*n* = 9/9) **(A)** and in aged compared to young microglial cells (*n* = 15/13) **(E)**. Macrophages from aged mice phagocytosed significantly less E. coli than macrophages from young mice after treatment with different concentrations of Pam_3_CSK_4_ **(B)**, LPS **(C)**, and CpG **(D)** (*n* = 6–9 per group). Similarly, microglial cells from aged mice phagocytosed less E. coli than microglial cells from young mice after treatment with a selected dose of Pam_3_CSK_4_ **(F)**, LPS **(G)**, and CpG **(H)** (*n* = 6 per group). Data are shown as means ± SD; *p < 0.05, **p < 0.01, ***p < 0.001, Student's *t*-test, Bonferroni correction in B, C, and D.

In the non-activated state, aged macrophages showed a significantly lower ability to phagocytose *E. coli K1* (38.6 ± 27.2 %) compared to young macrophages (100 ± 30.5 %, *p* = 0.0004; Figure 6A). The differences between aged and young macrophages concerning their phagocytic activity were even more pronounced after stimulation with the different TLR agonists. Aged macrophages phagocytosed significantly less *E. coli K1* compared to young macrophages after stimulation with Pam_3_CSK_4_ (0.01 μg/ml: *p* = 0.01; 0.1 μg/ml: *p* = 0.02; 1 μg/ml: *p* = 0.06; Figure 6B), LPS (0.0001 μg/ml: *p* = 0.24; 0.01 μg/ml: *p* = 0.008; 1 μg/ml: *p* = 0.03; Figure 6C), and CpG (0.1 μg/ml: *p* = 0.001; 1 μg/ml: *p* = 0.003; 10 μg/ml: *p* = 0.008; Figure 6D).

The results concerning the phagocytic ability of aged and young microglial cells were in accordance with the results obtained in macrophages. In the non-activated state, aged microglial cells showed a significantly reduced ability to phagocytose *E. coli* K1 (39.3 + 31.9 %) compared to young microglial cells (100 + 47.5 %, *p* = 0.0006; Figure 6E). The differences between aged and young microglial cells concerning their phagocytic activity were greater after stimulation with the different TLR agonists. Aged microglial cells phagocytosed less *E. coli* K1 compared to young microglia after stimulation with Pam_3_CSK_4_ (0.1 μg/ml: *p* = 0.01; Figure 6F), LPS (0.01 μg/ml: *p* = 0.06; Figure 6G), and CpG (1 μg/ml: *p* = 0.04; Figure 6H).

### Reduced release of NO by aged macrophages and microglial cells *in vitro* upon TLR activation

**Figure 7:**
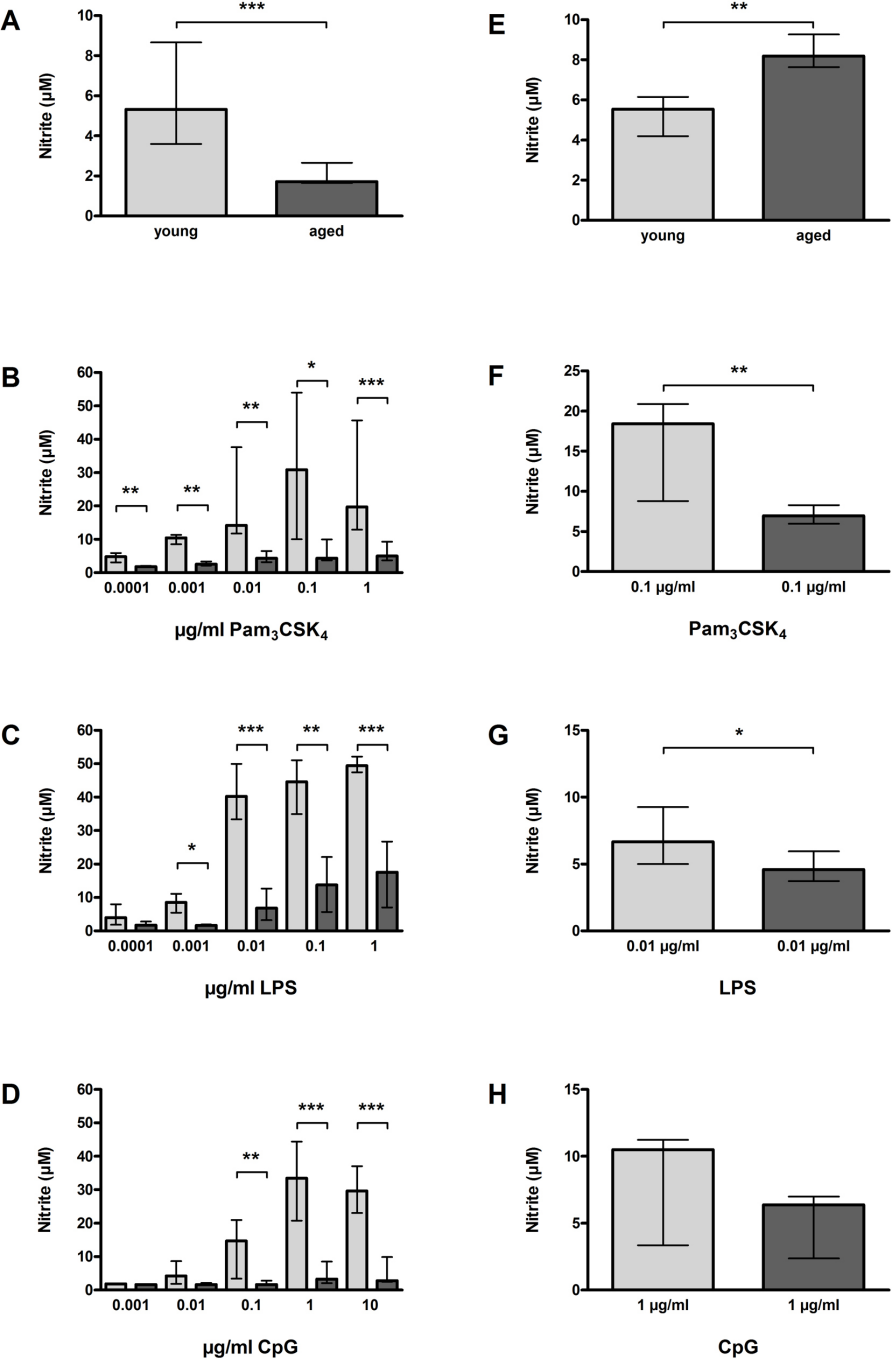
NO release by macrophages (A–D) and microglial cells (E–H) from young and aged mice *in vitro* In non-activated state, NO release was significantly lower in aged compared to young macrophages (*n* = 23/39) **(A)**, however, significantly higher in aged compared to young microglial cells (*n* = 15/14) **(E)**. Macrophages from aged mice released significantly less NO than macrophages from young mice after treatment with different concentrations of Pam_3_CSK_4_ **(B)**, LPS **(C)**, and CpG **(D)** (*n* = 6–18 per group). Similarly, microglial cells from aged mice released less NO than microglial cells from young mice after treatment with a selected dose of Pam_3_CSK_4_ **(F)**, LPS **(G)**, and CpG **(H)** (*n* = 6 per group). Data are shown as medians (25./75. percentile); *p < 0.05, **p < 0.01, ***p < 0.001, Mann-Whitney *U*-test, Bonferroni correction in B, C, and D.

In the non-activated state, NO release by aged macrophages [1.71 (1.66/2.66) μM] was significantly lower than NO release by young macrophages [5.32 (3.59/8.67) μM, *p* < 0.0001; Figure 7A]. In contrast, aged microglial cells released significantly more NO than young microglial cells in the non-activated state [8.19 (7.63/9.26) μM versus 5.54 (4.20/6.15) μM, *p* = 0.001; Figure 7E].

Upon stimulation with the three different TLR agonists, NO release by young macrophages increased in a dose-dependent manner, whereas NO release by aged macrophages remained on low levels. Thus, NO release of aged macrophages was significantly lower than that of young macrophages after stimulation with Pam_3_CSK_4_ (0.0001 μg/ml: *p* = 0.002; 0.001 μg/ml: *p* = 0.003; 0.01 μg/ml: *p* = 0.0005; 0.1 μg/ml: *p* = 0.02; 1 μg/ml: *p* = 0.0005; Figure 7B), LPS (0.001 μg/ml: *p* = 0.02; 0.01 μg/ml: *p* = 0.005; 0.1 μg/ml: *p* = 0.005; 1 μg/ml: *p* = 0.0005; Figure 7C), and CpG (0.1 μg/ml: *p* = 0.002; 1 μg/ml: *p* = 0.0005; 10 μg/ml: *p* = 0.0005; Figure 7D).

The results concerning the NO release of aged and young microglial cells upon stimulation with one representative concentration of each TLR agonists were in accordance with the results obtained in macrophages, however, here the differences between aged and young cells were less pronounced: Aged microglial cells released less NO than young microglial cells after treatment with 0.1 μg/ml Pam_3_CSK_4_ (*p* = 0.008; Figure 7F), 0.01 μg/ml LPS (*p* = 0.04; Figure 7G), and 1 μg/ml CpG (*p* = 0.21; Figure 7H).

### Reduced release of TNF-α by aged macrophages and microglial cells *in vitro* upon TLR activation

**Figure 8:**
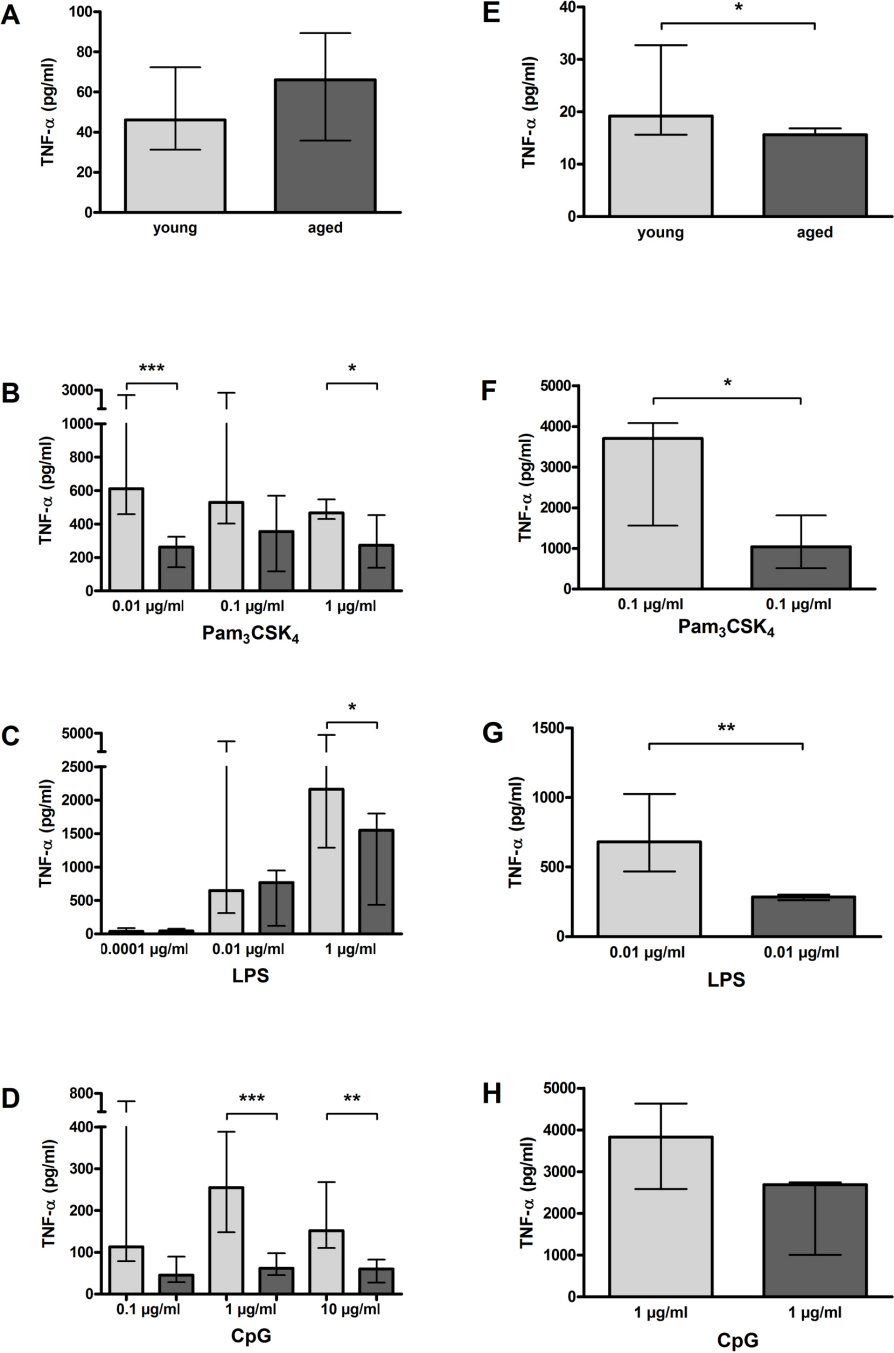
TNF-α release by macrophages (A–D) and microglial cells (E–H) from young and aged mice *in vitro* In non-activated state, TNF-α release form aged and young macrophages did not differ significantly (*n* = 14/21) **(A)**, however, it was slightly but significantly lower in aged compared to young microglial cells (*n* = 15/14) **(E)**. Macrophages from aged mice released less TNF-α than macrophages from young mice after treatment with different concentrations of Pam_3_CSK_4_ (*n* = 12–18) **(B)**, LPS (*n* = 6–15) **(C)**, and CpG (*n* = 9–15) **(D)**. Similarly, microglial cells from aged mice released less TNF-α than microglial cells from young mice after treatment with a selected dose of Pam_3_CSK_4_ (*n* = 6) **(F)**, LPS (*n* = 6) **(G)**, and CpG (*n* = 5) **(H)**. Data are shown as medians (25./75. percentile); *p < 0.05, **p < 0.01, ***p < 0.001, Mann-Whitney *U*-test, Bonferroni correction in B, C, and D.

In the non-activated state, the TNF-α release of aged and young macrophages did not differ [66.10 (35.80/89.40) pg/ml vs. 46.10 (31.30/72.35) pg/ml, *p* = 0.40; Figure 8A], and aged microglial cells released only slightly less TNF-α than young microglial cells [15.63 (15.63/16.48) pg/ml vs. 22.00 (15.63/51.40) pg/ml, *p* = 0.01; Figure 8E].

Aged macrophages released significantly lower amounts of TNF-α than young macrophages after stimulation with Pam_3_CSK_4_ (0.01 μg/ml: *p* = 0.0003; 0.1 μg/ml: *p* = 0.07; 1 μg/ml: *p* = 0.05; Figure 8B) and CpG (0.1 μg/ml: *p* = 0.09; 1 μg/ml: *p* = 0.0003; 10 μg/ml: *p* = 0.001; Figure 8D). After treatment with 1 μg/ml LPS, aged macrophages also released less TNF-α than young macrophages (*p* = 0.05), whereas there was no difference between aged and young macrophages concerning the TNF-α release after treatment with lower LPS doses (0.0001 μg/ml: *p* = 0.85; 0.01 μg/ml: *p* = 0.44; Figure 8C).

In accordance with the results in macrophages, aged microglial cells released lower amounts of TNF-α after stimulation with 0.1 μg/ml Pam_3_CSK_4_ (*p* = 0.05; Figure 8F), 0.01 μg/ml LPS (*p* = 0.002; Figure 8G), and 1 μg/ml CpG (*p* = 0.15; Figure 8H), with the differences reaching statistical significance in the cases of Pam_3_CSK_4_ and LPS.

### Reduced release of IL-6 by aged macrophages and microglial cells *in vitro* upon TLR activation

**Figure 9:**
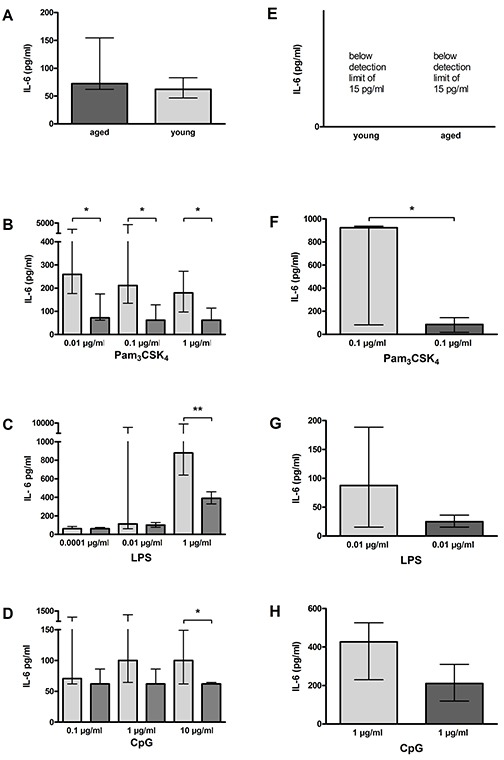
IL-6 release by macrophages (A–D) and microglial cells (E–H) from young and aged mice *in vitro* In non-activated state, IL-6 release form aged and young macrophages did not differ (*n* = 14/21) **(A)**. IL-6 concentrations in supernatants of aged and young macrophages were below the detection limit **(E)**. Macrophages from aged mice released less IL-6 than macrophages from young mice after treatment with different concentrations of Pam_3_CSK_4_ (*n* = 12–18) **(B)**, LPS (*n* = 6–15) **(C)**, and CpG (*n* = 9–15) **(D)**. Similarly, microglial cells from aged mice released less IL-6 than microglial cells from young mice after treatment with a selected dose of Pam_3_CSK_4_ (*n* = 6) **(F)**, LPS (*n* = 6) **(G)**, and CpG (*n* = 5) **(H).** Data are shown as medians (25./75. percentile); *p < 0.05, **p < 0.01, ***p < 0.001, Mann-Whitney *U*-test, Bonferroni correction in B, C, and D.

In the non-activated state, aged and young macrophages released comparable amounts of IL-6 [72.20 (62.00/154.50) pg/ml vs. 62.00 (46.60/83.03) pg/ml, *p* = 0.09; Figure 9A]. The IL-6 release of both aged and young non-activated microglial cells was below the detection limit of the ELISA (Figure 9E).

Aged macrophages released significantly lower amounts of IL-6 compared to young macrophages after stimulation with different doses of Pam_3_CSK_4_ (0.01 μg/ml: *p* = 0.03; 0.1 μg/ml: *p* = 0.01; 1 μg/ml: *p* = 0.01; Figure 9B], as well as after stimulation with 1 μg/ml LPS (*p* = 0.003; Figure 9C), and 10 μg/ml CpG (*p* = 0.04; Figure 9D). There were no statistically significant differences between aged and young macrophages concerning the IL-6 release after treatment with lower doses of LPS (Figure 9C) and CPG (Figure 9D).

Aged microglial cells released lower amounts of IL-6 after stimulation with 0.1 μg/ml Pam_3_CSK_4_ (*p* = 0.04; Figure 9F), 0.01 μg/ml LPS (*p* = 0.49; Figure 9G), and 1 μg/ml CpG (*p* = 0.10; Figure 9H), the difference, however, reached statistical significance in case of Pam_3_CSK_4_ only because of the high variation and the relatively low number of observations.

### Reduced release of KC by aged macrophages and microglial cells *in vitro*

**Figure 10:**
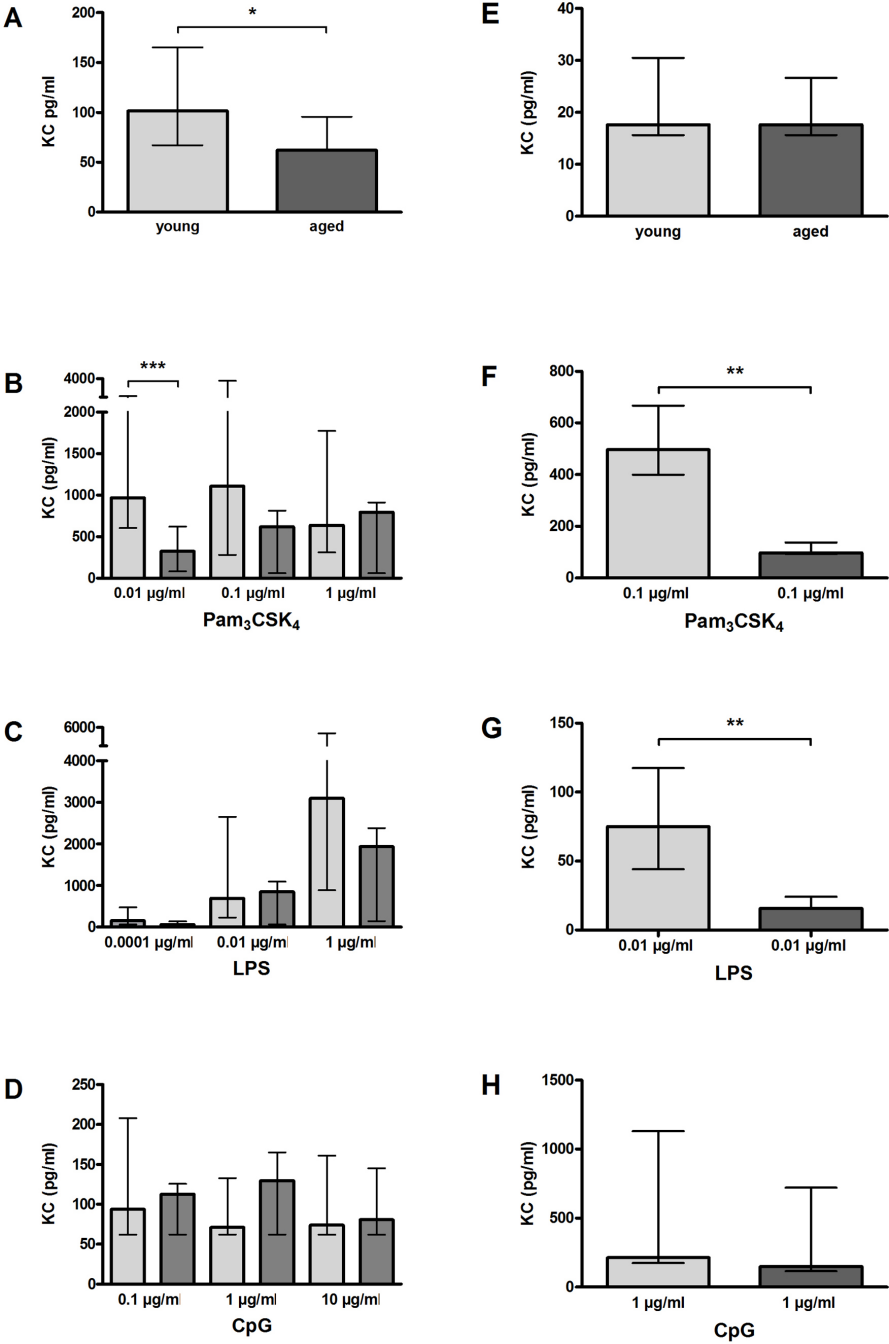
KC release by macrophages (A–D) and microglial cells (E–H) from young and aged mice *in vitro* In non-activated state, KC release from aged macrophages was significantly lower compared to aged macrophages (*n* = 14/21) **(A)**, however, it did not differ between aged and young microglial cells (*n* = 15/14) **(E)**. Macrophages from aged mice released less KC than macrophages from young mice after treatment with different concentrations of Pam_3_CSK_4_ (*n* = 12–18) **(B)** and LPS (*n* = 6–15) **(C)**. Similarly, microglial cells from aged mice released less KC than microglial cells from young mice after treatment with a selected dose of Pam_3_CSK_4_ (*n* = 6) **(F)** and LPS (*n* = 6) **(G)**. After stimulation with CpG, no differences concerning the KC release were observed between aged and young macrophages (*n* = 9–15) **(D)** and aged and young microglial cells (*n* = 5) **(H)**. Data are shown as medians (25./75 percentile); *p < 0.05, **p < 0.01, ***p < 0.001, Mann-Whitney *U*-test, Bonferroni correction in B, C, and D.

In the non-activated state, the KC release by aged macrophages was lower than by young macrophages [62.00 (62.00/95.80) pg/ml vs. 101.50 (67.33/165.20) pg/ml, *p* = 0.02; Figure 10A] whereas aged and young microglial cells released similar amounts of KC [16.61 (15.63/26.58) pg/ml vs. 17.60 (15.63/40.10) pg/ml, *p* = 0.68; Figure 10E].

After stimulation with 0.01 μg/ml Pam_3_CSK_4_, aged macrophages released significantly less KC than young macrophages (*p* = 0.0006), whereas KC release did not differ between aged and young macrophages after treatment with 0.1 μg/ml Pam_3_CSK_4_ (*p* = 0.07) and 1 μg/ml (*p* = 0.66; Figure 10B). After stimulation with 1 μg/ml LPS, KC release by aged macrophages tended to be lower than by young macrophages (*p* = 0.10), but it did not differ between aged and young macrophages after treatment with lower doses of LPS (Figure 10C). No differences were observed between aged and young macrophages concerning the release of KC after treatment with different concentrations of CpG (Figure 10D).

Aged microglial cells released significantly lower amounts of KC after stimulation with 0.1 μg/ml Pam_3_CSK_4_ (*p* = 0.003; Figure 10F) and 0.01 μg/ml LPS (*p* = 0.007; Figure 10G), whereas after treatment with 1 μg/ml CpG, KC release did not differ between aged and young microglial cells (*p* = 0.31; Figure 10H).

## DISCUSSION

Life-threatening infections are among the greatest challenges in geriatric medicine [3, 4, 31]. Clinical studies demonstrated that both the incidence and mortality of community- and hospital-acquired bacterial meningitis caused by different pathogens are strongly elevated in persons > 60 years compared to younger adults [5, 9, 6]. In a study from Pakistan, the relative frequency of *E. coli* meningitis was 7.3%, and mortality of *E. coli* meningitis was particularly high [32]. In mouse models, a higher age-related susceptibility to systemic infections has been observed with *S. pyogenes* [33] and *E. coli* [34]. Here, we established a geriatric mouse model for an intracerebral *E. coli* infection which reflects the clinical situation in aged patients with CNS infections: Aged mice showed a higher mortality (73%) than young mice (47%) after intracerebral injection of *E. coli* K1. Similar to the observation in the mouse model of *E. coli* sepsis [34], aged mice in our model showed an earlier development of clinical symptoms and a faster disease progression. Furthermore, aged mice had a more pronounced and sustained weight loss after intracerebral infection with *E. coli* comparable to aged rats after peripheral challenge with *E. coli* [35].

Our model tests the efficacy of the local and of the systemic defense against bacteria: after injection of rapidly multiplying pathogens in a small volume into the brain parenchyma, the immune response in the first hours executed by microglial cells and resident macrophages determines, whether the host organism survives or eventually succumbs to the infection. Circulating leukocytes generally need too much time to migrate into the central nervous compartments [36] and contribute to the elimination of bacteria in the first hours. Once a lethal infection is developing, the activity of circulating granulocytes and monocytes determines the bacterial concentrations in blood, spleen and partly also in brain [37].

The higher susceptibility of aged mice to the intracerebral *E. coli* infection was associated with a faster systemic spread of bacteria. Although they succumbed to infection earlier than young mice, at the time of death, aged mice had more than 100-fold higher *E. coli* concentrations in blood indicating an impaired leukocyte function, but similar concentrations of *E. coli* in brain. Similarly, after systemic infection with *S. pyogenes*, aged mice showed an impaired capacity to control bacterial growth in liver and spleen [33].

At 24 hours after intracerebral infection with a lethal dose of *E. coli*, levels of IL-6 and KC in cerebellar homogenates did not differ, however, serum levels of IL-6 and KC were significantly lower in aged than in young mice. Conversely, in some previous mouse models of systemic infection and sepsis, aged mice exhibited higher serum levels of pro-inflammatory cytokines than young mice [38, 33]. In old and young patients with systemic pneumococcal infections, concentrations of cytokines were similar on admission. However, the levels of TNF-α and soluble TNF receptor-1 one week after admission were higher in elderly (68–91 years) than in young (37–55 years) patients. This investigation in patients suggested that aging is associated with prolonged inflammatory activity reflecting a decreased ability to control the infection or a dysregulated cytokine response [39]. Our results indicate an impaired systemic cyto-/chemokine response of aged mice in the acute phase after intracerebral infection, which probably contributes to their reduced capacity to control systemic bacterial growth. They also show a delayed resolution of inflammation in old age as observed in humans: whereas inflammation rapidly declined in young surviving mice, inflammation in old mice persisted as illustrated by the higher meningeal inflammation score (Figure 4B) and number of Iba-1-positive microglia in surviving aged mice 15 days after infection.

Most of the young mice that survived the infection were able to clear the bacteria from the CNS and the periphery within 15 days, whereas in cerebellum and spleen of all but one aged mice bacteria were still detected at that time point. An inability to clear the *E. coli* infection also was observed in neutropenic mice [40]. However, we did not observe a substantial difference concerning the number of infiltrating leukocytes in the CNS after intracerebral infection with *E. coli* between aged and young mice. In both age groups, the number of infiltrating leukocytes strongly correlated with the number of bacteria. This suggests that leukocyte infiltration is not markedly impaired in aged mice, and reduced leukocyte recruitment is not the cause of their increased susceptibility to *E. coli* meningitis.

In CNS, meningeal and perivascular macrophages and microglial cells represent the first line of defense against bacteria. In the sepsis model of Goldmann et al., the reduced number of resident and peritoneal macrophages in aged mice was suggested to be the reason for the age-related increased susceptibility to *S. pyogenes* infection [33]. Others showed that the number of macrophages that could be derived from the bone-marrow of young and aged mice was similar [41]. Conversely, the number of macrophages we achieved by peritoneal lavage from aged mice was significantly higher compared to young mice. This suggests an elevated density of peritoneal macrophages in old age. Yet, we cannot exclude that the higher yield of the peritoneal macrophage preparation is a consequence of reduced adherence *in vivo*. We also evaluated the number of microglial cells in brains of young and aged mice after intracerebral *E. coli* infection: Microglia numbers were slightly higher in aged mice than in young mice (Figure 4F).

As we did not find a decreased number of immune cells in aged mice, we hypothesized that their increased susceptibility to the intracerebral *E. coli* infection is rather a consequence of impaired functions of the individual cells. Several studies have shown age-related defects in the functional capacity of macrophages and neutrophilic granulocytes [42, 43, 8]. To study whether age also affects microglial function, we prepared primary cultures of both peritoneal macrophages and microglial cells from aged and young mice: by light microscopy, no differences were detected between macrophages of young and aged mice or microglial cells of young and aged mice. Similarly, unstimulated macrophages derived from the bone marrow of aged and young mice showed no differences concerning size, degree of differentiation, DNA content and cell surface markers [41]. In our study, the ability to phagocytose *E. coli* was significantly impaired in aged macrophages and microglial cells in the non-activated state. After TLR activation by bacterial TLR agonists mimicking the situation during a bacterial infection, the difference between aged and young macrophages and microglial cells concerning their phagocytic activity was even greater. Similar to results from our previous experiments with microglial cells from newborn mice [15] and peritoneal macrophages from young adult mice, phagocytosis of *E. coli* by cells prepared from mice at the age of 2–3 months was strongly increased after stimulation with Pam_3_CSK_4_, LPS, and CpG. In contrast, phagocytic activity of aged cells was not stimulated by treatment with TLR2, 4 and 9 agonists. A reduced phagocytic activity of aged macrophages and microglial cells has been demonstrated in previous *in-vitro* studies: Wound macrophages from aged mice showed a reduced ability to phagocytose latex beads and opsonized sheep erythrocytes [44], and microglial cells isolated from brains of adult mice using different methods showed an age-related decline of the ability to phagocytose amyloid-beta [45, 46]. To our knowledge, this study is the first to demonstrate the defect of phagocytosis in macrophages and microglial cells prepared from old individuals by using live bacteria.

Confirming and extending data from Kissin et al. who observed a reduced NO release of splenic and peritoneal macrophages prepared from aged mice after stimulation with LPS [47], we observed a significantly reduced NO release of peritoneal macrophages from aged mice after stimulation with different concentrations of Pam_3_CSK_4_, LPS and CpG. Although in microglial cells stimulation could only be performed with selected concentrations of the TLR agonists because of the limited number of available cells, we could demonstrate that similar to peritoneal macrophages, NO release upon TLR activation was lower in aged than in young microglial cells.

Furthermore, we compared the release of the pro-inflammatory cytokines TNF-α and IL-6 and the chemokine KC by aged and young macrophages and microglial cells upon activation of TLR 2, 4 and 9. In accordance with results from previous *in-vitro* studies [48, 49, 8], aged peritoneal macrophages released significantly less inflammatory cyto-/chemokines than young macrophages upon TLR activation, probably because of age-related changes in the TLR signaling pathways [50]. The reduced release of cyto-/chemokines by aged macrophages could explain the lower serum levels of IL-6 and KC in aged mice after intracerebral *E. coli* infection which we found in our mouse model. As for phagocytic activity and NO release, we also found a similar behavior of aged microglial cells regarding the cyto-/chemokine release upon stimulation with TLR agonists: The release of TNF-α, IL-6 and KC was significantly reduced in aged compared to young microglial cells. Conversely, Frank et al. found an increased release of IL-1β and IL-6 by microglial cells prepared from the hippocampus of aged rats after stimulation with LPS [51]. Differences in the preparation process might be the reason for this discrepancy.

In conclusion, our results suggest that the age-related decline of microglia and macrophage functions, particularly the age-related decline of their phagocytic capacity, plays an essential role for the impaired elimination of bacteria and the higher mortality after an intracerebral bacterial challenge in aged mice. Our study identifies resident macrophages and microglial cells as potential therapeutic targets to improve the resistance of the aged host to CNS infections. Strategies to increase the phagocytic potential of aged macrophages and microglial cells appear promising for the prevention and therapy of CNS infections in elderly patients, yet, our *in-vitro* data suggest that many compounds suitable for this purpose in young mice may not work in old individuals. Moreover, the use of TLR agonists or other immunostimulants may entail the risk of inducing collateral damage to the nervous tissue [18, 19, 20, 37]. As a consequence of microglial diversity [52] compounds may be identified which increase phagocytosis of pathogens without collateral damage to the brain tissue [37]. Our geriatric mouse model of *E. coli* meningitis which mimics many aspects of the situation in humans appears suitable for investigating the beneficial and detrimental effects of immunomodulatory preventive or therapeutic strategies.

## EXPERIMENTAL PROCEDURES

### Bacteria

An *Escherichia coli* strain K1 (serotype O18:K1:H7) originally isolated from a child with meningitis was used for the *in-vivo* and *in-vitro* experiments [53, 15, 40].

### Mice

C57BL/6-N mice of different ages (Janvier, Le Genest Saint Isle, France) were used for the infection experiments and the preparation of primary cell cultures. Animal experiments were approved by the Animal Care Committee of the University Hospital of Göttingen, Germany, and by the Niedersächsisches Landesamt für Verbraucherschutz und Lebensmittelsicherheit (LAVES), Braunschweig, Lower Saxony, Germany.

### Intracerebral infection and monitoring of the disease course

All mice were anaesthetized by intraperitoneal injection of 2 mg ketamine and 0.2 mg xylazine before meningitis was induced by injection of 10 μl of 0.9% NaCl containing either 1 × 10^5^ or 7.5 × 10^5^ colony-forming units (CFU) of *E. coli* K1 into the superficial right frontal cortex and subarachnoid space through the right frontolateral skull. During the acute phase of the infection (up to 96 hours p.i.) mice were monitored every 12 hours, and then on day 7, 10, and 15 p.i.. Monitoring included weighing and assignment of a clinical score (0, no apparent behavioral abnormality; 1, moderate lethargy; 2, severe lethargy; 3, unable to walk; 4, dead) [54]. When a mouse was no longer able to walk (clinical score 3), it was sacrificed for ethical reasons. 15 days p.i., all surviving mice were sacrificed by cervical dislocation.

### Serum and tissue collection and sample processing

Blood was taken by retroorbital punction with a non-heparinized microhematrocrit capillary 24 hours p.i. (in a subset of animals) and directly before sacrificing. 10 μl of blood were directly diluted in saline (serial 1:10 dilutions) for determination of bacterial concentrations. The rest of the blood was collected in 1.5 ml Eppendorf tubes, stored at 4°C for 30 to 60 min to allow clotting, and centrifuged at 3000 × g for 10 min at 4°C. Then, serum was transferred to another tube and stored at –20°C until performance of ELISAs for measurement of cyto-/chemokine concentrations. After death as a consequence of infection or cervical dislocation, brains and spleens were removed. Half of the brain was fixated in 4% formaline and embedded in paraffine. 2 μm coronal brain sections were used for subsequent immunohistochemical analysis. One half of the cerebellum and of the spleen was homogenized in 500 μl saline, respectively.

### Determination of bacterial concentrations

To determine bacterial concentrations, 10 μl of serial 1:10-dilutions of blood, cerebellum and spleen homogenates in saline were plated on blood agar plates followed by incubation for 24 hours at 37° C and 5% CO_2_. The detection limit of this method was 1000 CFU/ml blood or tissue. For mice that survived the infection and were sacrificed 15 days p.i., the detection limit was reduced to 100 CFU/ml cerebellum and spleen, respectively, by plating 100 μl of tissue homogenates on blood agar plates.

### Primary murine peritoneal macrophage and primary murine microglia cell cultures

For preparation of primary microglial cultures and peritoneal macrophages, C57BL/6-N mice at the age of 2 months and 18 months were used. From each mouse, both peritoneal macrophages and microglial cells were prepared after anaesthesia with diethyl ether and decapitation. Cells were cultured in DMEM with Glutamax I (Gibco) supplemented with 10% FCS, 100 U/ml penicillin and 100 μg/ml streptomycin. Cell culture plates and 75 cm^2^ cell culture flasks from Corning Costar (Wiesbaden, Germany) were used. Incubation was performed at 37°C and 5% CO_2_ in a humidified atmosphere.

Peritoneal macrophages were harvested by peritoneal lavage with pre-cooled phosphate-buffered saline (PBS; 5 × 1 ml). Cells were collected by centrifugation (1000 × g, 10 min, 4°C), and the pellet was resuspended in cell culture medium. Cells were plated in 96-well cell culture plates at a density of 50000 cells/well. After 2 hours, culture medium was changed completely. After incubation over night, macrophages were used for subsequent experiments.

Microglial cells were prepared and cultivated using a protocol for primary microglial cultures of adult mice [55]. Briefly, after careful removal of the meninges, the brain was mechanically dissociated and washed in DMEM followed by enzymatic processing with trypsine and desoxyribonuclease I. After centrifugation at 250 × g and 4°C for 10 min, the cell pellet was resuspended in cell culture medium and passed through a 40 μm cell filter (VWR, Darmstadt, Germany). Then, the cell suspension was seeded on two 75 cm^2^ cell culture flasks containing confluent astrocyte layers that previously had been established from newborn C57BL/6-N mice [13] and cleared from newborn microglial cells by treatment with clodronate (200 μg/ml; Merck, Darmstadt, Germany) for 3 days. Cell culture medium was changed completely every 3–4 days. After 14 days, adult microglial cells were separated from the astrocyte layers by shaking 200 x/min for 30 min. The supernatant was collected, and fresh cell culture medium was added to the astrocyte layers for further cultivation of remaining microglial cells. Microglial cells in the supernatant were isolated by centrifugation (250 × g, 10 min, 4°C), and the pellet was resuspended in cell culture medium. Cells were plated in 96-well cell culture plates at a density of 50000 cells/well. After incubation over night, microglial cells were used for the subsequent experiments.

### Stimulation of macrophages and microglial cells with TLR agonists

Cultured cells were treated with different concentrations of TLR agonists for 24 hours in the presence of interferon-γ (100 U/ml; Sigma, Taufkirchen, Germany). Tripalmitoyl-S-glyceryl-cysteine (Pam_3_CSK_4_; EMC Microcollections GmbH, Tübingen, Germany) was used as specific agonist of TLR1/2. For activation of TLR4, microglial cells were exposed to endotoxin (LPS) from *Escherichia coli* serotype 026:B6 (Sigma, Taufkirchen, Germany). CpG oligodesoxynucleotide (ODN) 1668 (TCC ATG A**CG** TTC CTG ATG CT) from TIB Molbiol (Berlin, Germany) was used as specific ligand of TLR9. Unstimulated cells were treated with cell culture medium containing 100 U/ml interferon-γ. After 24 hours of stimulation, supernatants were stored at –80°C until measurement of NO, cytokine and chemokine levels. Cells were used in the bacterial phagocytosis assay. Cell viability was determined using the WST-1 Cell Proliferation Reagent (Roche Applied Science, Mannheim, Germany) according to the manufacturer's instructions.

### Phagocytosis assay

Phagocytosis assays were performed as previously described [15]. After stimulation, macrophages or microglia (50000 cells/well) were exposed to 5 × 10^6^ colony forming units (CFU) *E. coli* K1/well (100 bacteria per phagocyte) for 90 minutes. Then, extracellular bacteria were killed by treatment with 100 μg/ml gentamicin (Sigma, St. Louis, MO, USA) for 60 minutes. Cells were lysed with distilled water, and the number of intracellular bacteria was determined by quantitative plating of serial 1:10-dilutions on blood agar plates.

### Quantification of TNF-α, IL-6, KC, and NO

Concentrations of TNF-α, IL-6, and KC (CXCL1) in mouse serum, cerebellum homogenates, and in cell culture supernatants were measured by ELISA. TNF-α levels were determined using antibody pairs from BioLegend (Biozol, Munich, Germany), and DuoSet ELISA Development Kits (R&D Systems, Wiesbaden, Germany) were used for the measurement of IL-6 and KC. NO release of macrophages and microglial cells *in vitro* was assessed by the measurement of nitrite, one of its stable reaction products, in the cell culture supernatants using the Griess reagent [13].

### Stainings

Isolectin B4 staining [13] was used to assess the purity, density and morphology of macrophages and microglial cells *in vitro*. In each experiment, seeded cells were stained and counted in representative wells in order to assure equal cell numbers.

Chloroacetate esterase (CAE) stainings (Naphthol-AS-D-chloracetate esterase Kit, Sigma-Aldrich, Germany) were performed on brain sections to evaluate inflammation in three superficial meningeal regions and the hippocampal fissure [40]. CAE staining is a method to detect neutrophilic granulocytes. Stained sections were semi-quantitatively scored by a blinded investigator for the number of CAE-stained leukocytes in one high-power field (×40 objective) per region: no leukocytes (0), <10 leukocytes (1), 10–50 leukocytes (2), >50 leukocytes (3). For each animal, the scores of the individual fields were added and then divided by the number of scored regions.

Ionized calcium-binding adaptor molecule 1 (Iba-1) was used to identify microglial cells. In each animal, Iba-1-positive cells were quantified in at least three different neocortical regions (x20 objective), added, and then divided by the number of scored regions [40].

### Statistics

GraphPad-Prism-software 5.0 (GraphPad-Software, San Diego, California, USA) was used for statistical analyses and graphical presentation. Parametric data (weight, numbers of Iba-1^+^ cells, and numbers of phagocytosed bacteria) were expressed as means ± standard deviations (SD) and compared using the Student's *t*-test. Non-parametric data (clinical score, bacterial concentrations in serum and tissue, cyto-/chemokine concentrations, NO concentrations, meningeal inflammation score) were expressed as medians and interquartile ranges and compared using the Mann-Whitney *U*-test. The Bonferroni method was applied in case of multiple comparisons to correct for repeated testing. Log-rank test was performed for the comparison of survival curves. Correlations between bacterial concentrations in cerebellum and the number of microglial cells/the meningeal inflammation score were analyzed using Spearman's rank correlation coefficient (r_S_). For all analyses, *p* values ≤0.05 were considered statistically significant.
